# Conditional Granger Causality Analysis of Effective Connectivity during Motor Imagery and Motor Execution in Stroke Patients

**DOI:** 10.1155/2016/3870863

**Published:** 2016-04-20

**Authors:** Li Wang, Jingna Zhang, Ye Zhang, Rubing Yan, Hongliang Liu, Mingguo Qiu

**Affiliations:** ^1^Department of Medical Image, College of Biomedical Engineering, Third Military Medical University, No. 30, Gaotanyan Street, Shapingba District, Chongqing 400038, China; ^2^Department of Rehabilitation, Southwest Hospital, Third Military Medical University, No. 30, Gaotanyan Street, Shapingba District, Chongqing 400038, China

## Abstract

*Aims.* Motor imagery has emerged as a promising technique for the improvement of motor function following stroke, but the mechanism of functional network reorganization in patients during this process remains unclear. The aim of this study is to evaluate the cortical motor network patterns of effective connectivity in stroke patients.* Methods.* Ten stroke patients with right hand hemiplegia and ten normal control subjects were recruited. We applied conditional Granger causality analysis (CGCA) to explore and compare the functional connectivity between motor execution and motor imagery.* Results.* Compared with the normal controls, the patient group showed lower effective connectivity to the primary motor cortex (M1), the premotor cortex (PMC), and the supplementary motor area (SMA) in the damaged hemisphere but stronger effective connectivity to the ipsilesional PMC and M1 in the intact hemisphere during motor execution. There were tighter connections in the cortical motor network in the patients than in the controls during motor imagery, and the patients showed more effective connectivity in the intact hemisphere.* Conclusions.* The increase in effective connectivity suggests that motor imagery enhances core corticocortical interactions, promotes internal interaction in damaged hemispheres in stroke patients, and may facilitate recovery of motor function.

## 1. Introduction

Stroke patients often exhibit a variety of impairments in motor ability [1]. These motor deficits are typically treated with physical therapy. However, current physical treatment methods lack patient activity, which impacts the outcome of treatment. A new approach is therefore needed to remedy this deficiency of physical therapy. Functional recovery in stroke patients is achieved largely through reorganization processes in the damaged brain, and studies have suggested that repetitive mental practice could cause plastic changes in the brain [2–4]. To this end, motor imagery has emerged as a promising technique to improve motor function. Motor imagery is a cognitive rehearsal of physical movements that is defined as the internal reactivation of any first-person motor program without overt motor output [5, 6]. Some studies have used motor imagery as an auxiliary treatment in combination with physical therapy, particularly in the treatment of upper limb impairment after stroke [7, 8]. A few groups have demonstrated that motor imagery plays an important role in the recovery of motor abilities in patients with movement disorders [9–11]. Functional imaging studies have shown that the primary motor cortex (M1), the premotor cortex (PMC), and the supplementary motor area (SMA) were all activated during both motor execution and motor imagery tasks and that the activation of brain regions upon motor execution and motor imagery exhibited a large overlap [12, 13].

Neural reorganization depends on the information provided by sensorimotor efferent-afferent feedback loops [14]. Therefore, elucidation of neural connectivity is important for understanding the mechanisms underlying motor control and motor recovery. Although functional imaging studies have found that motor imagery activates the motor area in stroke patients, the information exchange mechanism in these areas is not clearly understood. Recently, there have been several studies investigating direct causality among different activated brain regions in stroke patients using different analytical methods for fMRI data. Sharma et al. analyzed the interactions between brain regions by structural equation modeling (SEM) of the fMRI signal in stroke patients and observed abnormal corticocortical connectivity in the motor system after subcortical stroke, even after significant recovery [7]. Bajaj et al. used a dynamical causal modeling (DCM) approach to task-based fMRI data to study brain effective connectivity within motor networks of stroke patients and found that PMC and M1 play a crucial role during motor imagery, as well as during motor execution [15]. However, these methods have some limitations. SEM is not a time series model and can even randomly change the data in a time sequence; because the same result can be obtained using model analysis, SEM analysis is more suitable for PET data. DCM is dependent on the selection of an interaction area in advance and assumes that there is an influence between the two regions. If the area selection and estimation of the influence on the connected brain area are not accurate, the final result will lead to incorrect conclusions.

Granger causality analysis (GCA) was formalized in the context of linear vector autoregression (VAR) models in 1969. A time series *x* is said to “G-causes” *y* if *y* can be better predicted using the past values of both *y* and *x* (full model) compared with the prediction of *y* using only the past values of *y* (restricted model) [16]. Without* a priori* knowledge of the directional connectivity among brain regions, GCA is better suited than SEM and DCM methods for the study of direct interactions between brain regions [17, 18]. However, GCA does not make use of multivariate data and can only address bivariate time series. The conditional Granger causality analysis (CGCA) extends Granger's original definition of causality to multivariate cases, the linear direct influence from *x* (origin) to *y* (target) conditional on *z* (*F* *x* → *y*∣*z*). Each simultaneous time series is chosen alternatively as the origin or target. When two of the series were chosen as the origin and target, the remaining series were composed of *z* values and served as conditions of the CGC analysis. Thus, we used CGCA to analyze fMRI data and could examine abnormal interactions among motor networks in stroke patients to better understand the neurobiological basis of these disorders.

In the present study, we used CGCA analysis of fMRI data to investigate the effective connectivity patterns of motor networks during motor execution and motor imagery in stroke patients and age-matched normal controls. We hypothesized that (1) the effective connectivity in the cortical motor network would be decreased in patients compared with controls due to brain damage; (2) the same changes in effective connectivity would be found in those patients whose motor network was changed during motor imagery; and (3) interactions within regions restricted to the motor network would reveal a specific compensatory mechanism in stroke patients that affected the motor network.

## 2. Materials and Methods

### 2.1. Subjects

Twelve stroke patients with right hand hemiplegia were prospectively recruited (50.60 ± 10.80 years). A stroke neurologist selected stroke patients by magnetic resonance imaging and Fugal-Meyer Assessment (FMA) motor function scores, which required initial disease occurrence and obvious right hand hemiplegia. All stroke patients had only unilateral left brain damaged. The specific inclusion criteria included extension of the ipsilateral wrist >10° and extension between the thumb, the metacarpophalangeal joint, and the interphalangeal joints of at least two fingers >10°. Ten age-matched control subjects (50.10 ± 16.20 years) were recruited through a local advertisement. The control subjects had no history of medical disorders and did not regularly take medication. All of the subjects were right-handed. Handedness was based on the hand currently used for lateralized tasks, such as writing. Written consent was obtained from each participant, and the protocol was approved by the Ethics Committee of the Third Military Medical University.

For each subject, mental status was assessed by the Mini-Mental State Examination (MMSE), and motor imagery performance was evaluated by the Movement Imagery Questionnaire, Revised Second Edition (MIQ-RS) [19]. A MIQ-RS score below 28 indicates that subjects unable to adequately complete the MI were excluded. Fugal-Meyer Assessment (FMA) [20] motor function scores were applied in the patient group.

### 2.2. Scanning Procedures

The experiment was performed using a 3.0 T MRI scanner (Trio, Siemens Medical Erlangen, Germany) using a gradient-recalled echo planar imaging (EPI) sequence. The acquisition parameters were as follows: TR = 2000 ms, TE = 30 ms, flip angle = 90°, 64 × 64 voxel matrix, FoV = 220 mm, 27 contiguous axial slices acquired in interleaved order, and thickness = 4.0 mm. High-resolution T1-weighted structural images were also acquired using the 3D MP-RAGE pulse sequence with the following parameters: TR = 1900 ms, TE = 2.52 ms, flip angle = 15°, voxel matrix = 256 × 256 and FoV = 240 mm, 176 contiguous axial slices, and thickness = 1.0 mm.

### 2.3. Experiment

The fMRI experiment performed in our previous study was repeated in the present study [21]. All subjects experienced 2 sequential runs: right hand motor execution and right hand motor imagery. Each run included five stimulation blocks, and each block lasted 60 seconds, including 30 seconds for motor execution/motor imagery and 30 seconds for the rest. During the motor execution run, the screen presented a picture of the corresponding finger movement at a frequency of 1 Hz. During the motor imagery session, the screen presented the direction indicated by the corresponding arrowhead: the left arrow indicates the motor imagery of the left hand and the right arrow indicates the right hand. During the rest period, the black central cross on the screen reminded participants to place their hands on the sides of the body and to breathe quietly [21]. We apply the calibrated bilateral fiber optic-gloves to monitor the participants' behavior during the entire process of the experiment (Fifth Dimension Technologies, Pretoria, South Africa).

### 2.4. Data Preprocessing

The experimental data were preprocessed using statistical parametric mapping software (SPM8, http://www.fil.ion.ucl.ac.uk/spm/software/spm8/). All the 150 images were corrected for the acquisition time delay among different slices and realigned to the first volume for head motion correction. Two stroke subjects whose head motion was greater than 2 mm or whose rotation was greater than 5° were excluded from the subsequent analysis. Then all the realigned images were spatially normalized into a standard stereotaxic space with voxel size of 2 × 2 × 2 mm^3^ using the Montreal Neurological Institute (MNI) EPI template; the voxel coordinates were transformed from the MNI coordinates to coordinates. They were spatially smoothed using a Gaussian kernel with an FWHM of 6 mm; task-related *t*-contrast images were calculated for each subject by using the *t*-statistic. Thereafter, one-sample *t*-test was performed on all the individual contrast images for the group analysis of motor execution and motor imagery tasks, respectively.

### 2.5. Identification of Regions-of-Interest (ROIs)

Our previous studies demonstrated that SMA, PMC, and M1 are critical regions in both motor execution and motor imagery tasks [21]. Therefore, we selected these regions as ROIs. The peak voxels and ROI of the subject were determined by individual SPM(*t*) maps. The ROI of each subject was a radius of 6 mm in the activated area, which was restricted to the activated voxels detected by the group analysis. The center of each ROI was at the highest positive *t* value.

### 2.6. Effective Connectivity among ROIs

Given three vector stochastic (random) processes *X*, *Y*, and *Z*, the CGC from *Y* to *X* given *Z* is calculated by combining two multivariate autoregressive estimations. In this study, CGCA analysis was implemented using the “GCCA” toolbox in MatLab [22, 23]. The time-course of one ROI is associated with *X*, and another is associated with *Y*. *Z* represents all remaining ROI time-courses other than *X* and *Y*. CGCA was performed to test the causal influences among ROIs. The order of the autoregressive model was estimated to use the Bayesian Information Criterion. The coefficients of the models were calculated using a standard least squares optimization. To assess the statistical significance of the Granger causality results, the threshold for significance was set at *p* < 0.05 for each subject and the group analysis was performed based on nonparametric statistical analyses and the Wilcoxon signed-rank test (*p* < 0.01). To better evaluate the causal interactions among the nodes of the motor network, we implemented a two-sample *t*-test between the two groups (Wilcoxon signed-rank test, *p* < 0.05).

## 3. Results

### 3.1. Behavioral Data

In the patient group, the mean imagination score was 37.8 ± 5.53, the mean MMSE score was 27.00 ± 1.80, and the mean FMA score was 37.00 ± 7.60.  Table 1 summarized the demographic and information about stroke patients. In the control group, the mean imagination score was 39.90 ± 2.73, and the mean MMSE score was 28.40 ± 1.35. The MMSE scores were not significantly different between the two groups (*p* = 0.07). All subjects had an imagination score greater than 28 and exhibited movement imagination ability, which was consistent with the experimental requirements [19]. There was no significant difference in the imagination score between the two groups. The final data included 10 stroke patients and 10 healthy subjects.

### 3.2. Brain Activation

**Figure 1:**
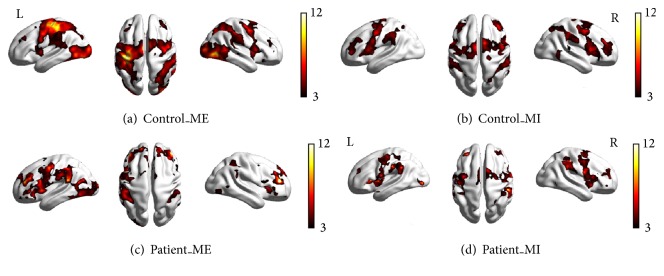
Brain activation in the control and patient groups under different conditions. (a) Control subjects during motor execution; (b) controls during motor imagery; (c) patients during motor execution; (d) patients during motor imagery. All voxels were significant at *p* < 0.01, corrected for FDR at the whole-brain level.

**Figure 2:**
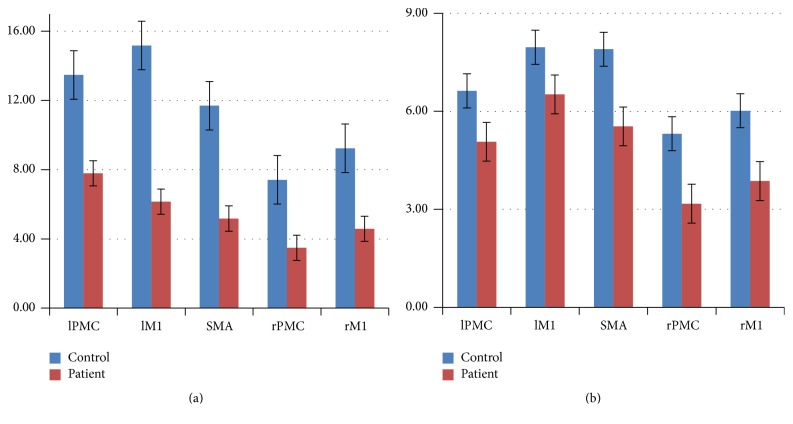
The statistical analysis of mean value of the highest positive *t* value of the subject-specific ROI in the two groups, (a) motor execution; (b) motor imagery. lPMC = left premotor cortex; lM1 = left primary motor cortex; SMA = supplementary motor area; rPMC = right premotor cortex; rM1 = right primary motor cortex.

As shown in Figures 1(a) and 1(b) and Figures 2(a) and 2(b), in the control group, the cortical motor areas were activated during both motor execution and motor imagery, including significant activation of the M1, PMC, and SMA, and stronger activation in the contralateral cortical motor areas than in the ipsilateral brain areas was noted. However, the intensity and area of activation were reduced during motor imagery (Table 2).

In the patient group, the contralateral activated cortical motor areas were significantly reduced compared with the controls during motor execution (Figures 1(c), 1(d), and 2). However, the intensity and activation area were not significantly reduced during motor imagery.

### 3.3. Effective Connectivity in the Control Group

**Figure 3:**
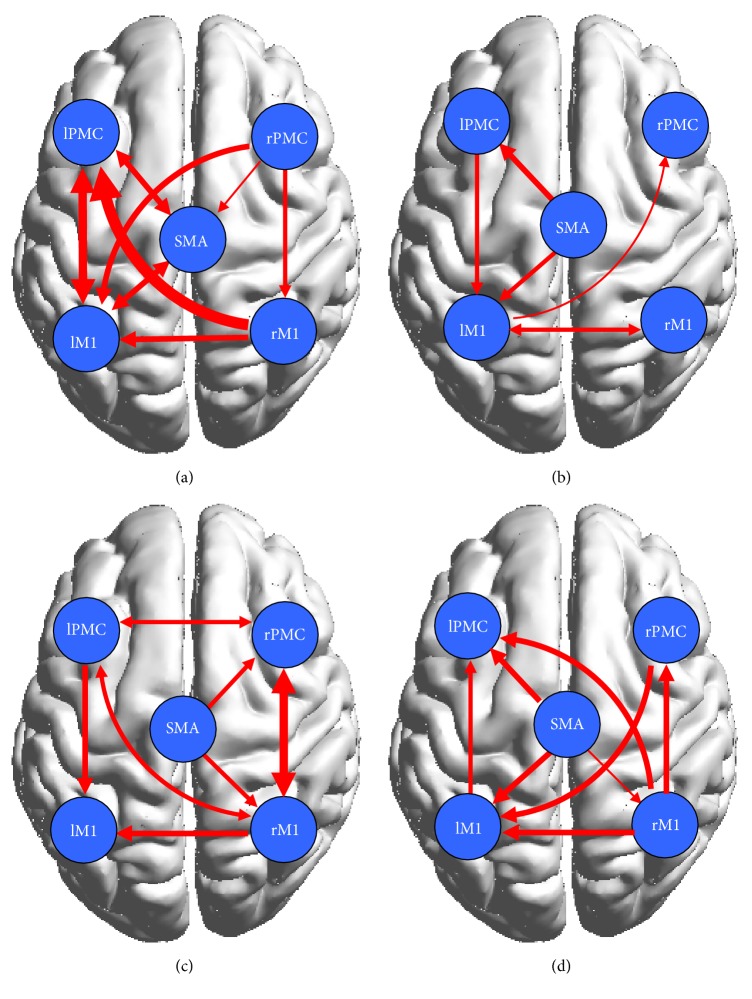
(a) and (b) display significant effective connectivity within the cortical motor network in the healthy controls during ME (a) and MI (b). (c) and (d) display significant effective connectivity within the cortical motor network in the patients during ME (c) and MI (d). The thickness of the lines is proportional to the connection strength.

The network showed strong effective connectivity among the contralateral regions both during motor execution and motor imagery (Figures 3(a) and 3(b)). The connections were decreased to a larger extent during motor imagery than during motor execution with respect to intensity and quantity, and effective bilateral connectivity was observed among the contralateral regions during motor execution, but such connections were not observed during motor imagery.

### 3.4. Effective Connectivity in the Patient Group

We observed decreased effective connections in damaged brains during motor execution, but there were more effective connections in the intact brains during motor execution because there was an information loop between the SMA and the ipsilateral PMC and M1. We observed a more complex effective connective motor network during motor imagery in the patients as there was an information loop in the damaged brains and interaction between the bilateral PMC and the M1 during motor imagery (Figures 3(c) and 3(d)).

### 3.5. Between-Group Effective Connectivity

**Figure 4:**
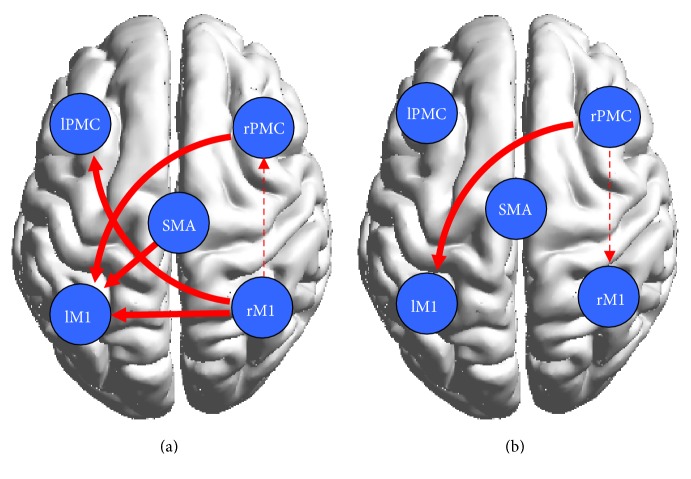
Results of the motor network causal interactions in the controls versus the stroke patients during ME and MI. Solid lines indicate significantly stronger connections in the control group than in the patient group, and dashed lines indicate the opposite.

To better evaluate the causal interactions within the motor network, we implemented a two-sample *t*-test between the two groups (Figures 4(a) and 4(b)). The motor network exhibited stronger interactions in the healthy controls during motor execution, as was the case between the right PMC and the left M1, as well as the right M1 and the left PMC and M1 (solid line). However, the interactions between the left M1 and the PMC in the control subjects were weaker than those in the stroke patients (dashed line), as was the case with the effective connections from the ipsilateral M1 to the PMC. There was little difference in the motor networks of both groups during MI. The effective connection from the right PMC to the left M1 (solid line) was stronger during motor imagery in the controls than in the stroke patients; however, the effective connection from the left M1 to the PMC (dashed line) was weaker in the controls than in the stroke patients.

### 3.6. The Statistical Analysis of All the In and Out Degrees

In order to more clearly know about the role of motor areas during tasks, we calculated the network parameters degree (defined as the number of edges connected to the node) and analyzed every ROI of all the in and out degrees. In Figure 4, left brain areas revealed higher degree in the control group than in the patient group during motor execution; however opposite results were shown in the right brain. The right brain area revealed lower degree in the control group than in the patient group during motor imagery.

## 4. Discussion

In our study, the bilateral PMC, M1, and SMA were significantly activated during both motor execution and motor imagery in the controls. These areas are involved in general motor control and in postural control of the wrist, in particular [24–26]. In the patients, the activation of these areas was more extensive. In addition to the obvious activation of the motor area, the frontal lobe and parietal lobe were also significantly activated. This finding indicates that the patients had motor difficulty and needed to recruit more brain areas to complete tasks. Interestingly, greater activation of cortical motor areas was observed in the patients during motor imagery than during ME, including in the bilateral PMC, M1, and SMA. This activation confirmed that motor imagery represents an effective means to stimulate brain regions that are normally involved in the planning and control of movement of affected limbs [25, 27].

CGCA was employed to measure the influence of one time series on another time series in the presence of a third. However, until now, there have been few attempts to investigate interactions within motor regions using GCCA in conjunction with fMRI. This paper thus aimed to explore the interactions in the cortical motor network in stroke patients during both ME and MI using CGCA. We found that effective connections in the stroke patients were significantly decreased in the lesioned hemisphere compared with the control subjects during ME. Some connections were disrupted because of a brain lesion. Previous studies on stroke have reported that functional connectivity was decreased in the motor network and that abnormal connectivity has been interpreted to be directly related to brain lesions [28]. Our results from CGCA were consistent with these findings and confirmed our hypothesis that effective connectivity in the cortical motor network would be decreased in the patients compared with the controls. In Figure 3(a), there was bidirectional connection between the LPMC and LM1 in the control group; however, only unidirectional connection from LPMC to LM1 was shown in the patient group (Figure 3(c)). The disappearance of connection from LM1 to LPMC may indicate that LM1 had no impact on LPMC because of the lesion in the patient group. The reduced connectivity in the motor network leads to poor information integration in the brain, resulting in difficultly with movement control in the patients.

The two-sample *t*-test between the groups revealed that the interaction of the two hemispheres was weaker during motor execution in the patient group than in the control group and that the connectivity from the left M1 to the right PMC, as well as the connectivity from the left PMC to the right M1, was stronger in the controls. Ferbert et al. reported that hemispheric interactions are normally important for the coordination of hand movements [29]. In our study, the weak interaction between the two hemispheres in the patients resulted in looser connections within brain networks compared with the controls. Brain networks are less efficient in stroke patients compared with healthy people, which may explain why it is more difficult for stroke patients to perform movements. In contrast, the effective connectivity was stronger for the ipsilesional hemispheric connections during ME in the patient group than in the control group, as was the connectivity from the ipsilesional PMC to the M1. As other studies have reported, stroke patients activate more ipsilesional motor brain areas to perform simple hand movements using the affected arm [30–33]. In our study, the same results were obtained, and the ipsilesional regions were significantly activated. In addition, we observed more effective connectivity in the ipsilateral hemisphere during motor execution in the stroke patients. This may be because lesions can cause brain functional reorganization, requiring patients to recruit more resources in the intact hemisphere to complete motor tasks.

In our study, we found great similarity in the cortical motor network during motor imagery in both groups. A loop of effective connectivity was observed within the SMA and contralateral M1 and PMC, and strong coupling between the bilateral PMC and M1 was observed for both groups. In addition, there were tighter connections within the cortical motor network during motor imagery in the patients than in the controls. In contrast to the effective connectivity in the cortical motor network in patients during motor imagery and motor execution, the interactions among these regions were enhanced during motor imagery in a manner similar to the controls. Pool et al. found that stronger coupling between the contralateral SMA/PMA and the M1 could enable increased motor performance in unilateral hand movements in healthy people [34]. In our study, there was no such coupling within the contralateral regions in the patients during motor execution, which may be related to brain damage; however, the interactions were observed during motor imagery in the patients. These results indicate that although the lesioned hemisphere will cause abnormal network activity during motor execution in patients, it has no obvious effect on motor imagination. These findings suggested that our second hypothesis is false. The increased effective connectivity suggested that motor imagery enhanced corticocortical interactions; in particular, it promoted internal interaction in the damaged hemispheres in stroke patients and may have facilitated recovery of motor function. These results explain the function of motor imagery in the rehabilitation of patients with stroke to a certain extent.

Although the two groups of human motor networks exhibited great similarity during motor imagery, significant differences in effective connectivity were also observed. The stroke patients showed more effective connectivity in the intact hemisphere. In the two-sample *t*-test between the two groups, we found that ipsilesional PMC and M1 increased connections in the patient group. Sharma et al. found that coupling between the ipsilesional PMC and the M1 was increased during motor imagery tasks, thereby enhancing cortical–cortical interactions [7]. Our study confirmed the changes of effective connectivity within the intact hemisphere by CGCA and suggested that motor imagery could increase interactions within intact brain. Furthermore, lesions were likely to cause the reorganization of the motor system and to alter motor imagery-related neurological function [7, 35]. In another study, the effective connectivity from the ipsilesional PMC to the contralateral M1 was weaker in patients than in controls. The decreased coupling between the two hemispheres suggested that although motor imagery could drive the interaction between the two hemispheres in the patients, the connectivity strength was weaker than in the controls. Thus, the lesion may lead to increased difficulty with integrating information and controlling motion.

**Figure 5:**
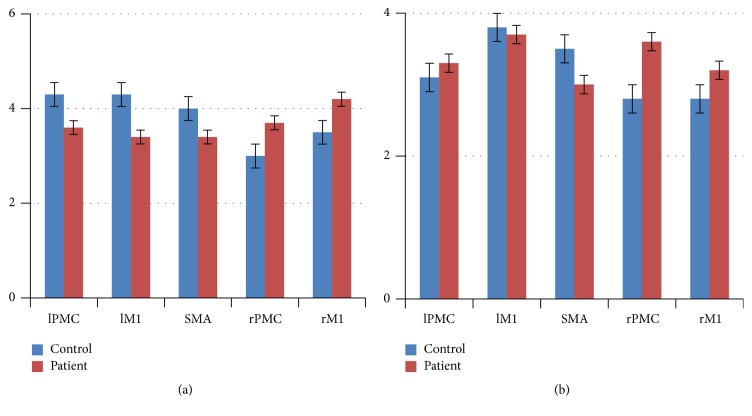
The statistical analysis of the all in and out degrees in each ROI of both groups during tasks, (a) motor execution; (b) motor imagery.

In the brain network, the degree of ROI indicates its centricity and importance. As shown in Figure 5(a), ROI had a higher degree in the contralateral hemisphere than in the ipsilateral hemisphere during right hand motor execution in the control group, while the patient group had opposite results. These suggested that the lesions were affecting the centricity of the contralateral hemisphere. The increased degree of ipsilateral brain regions may be a compensation for brain injury during motor execution in the patient group.  Figure 5(b) showed that the centricity and importance of the ipsilateral hemisphere was also improved during movement imagery in patients group.

Our study has some limitations. First, the sample size was limited. Second, we used CGCA and found that the motor network changed in stroke patients. These results were limited to the motor cortical region and did not involve subcortical areas. Many subcortical regions are known to be involved in movement, especially in stroke patients, because of the reorganization of brain function. Our next study will attempt to expand the research sample size and to further study the functional connectivity of the subcortical regions to more clearly understand the mechanisms of functional network reorganization in stroke patients.

## 5. Conclusions

In conclusion, the results of the current CGCA study describe the effective connectivity patterns in the cortical motor network after stroke during motor execution and motor imagery tasks. Effective connections in the stroke patients were significantly decreased in the lesioned hemisphere compared with the healthy controls during motor execution but were increased in the ipsilateral hemisphere compared with the controls during motor execution. This demonstrated that these patients recruit resources in the intact hemisphere to fulfill motor tasks. There were tighter connections in the cortical motor network in the patients than in the controls during motor imagery, and the patients showed more effective connectivity in the intact hemisphere. These results indicate that although the damaged hemisphere will cause abnormal motor network activity during motor execution in patients, it has no obvious effect on mental practice. The increase in effective connectivity suggests that MI may enhance interregional interaction and facilitate recovery of motor function.

## Figures and Tables

**Table 1:** Patient demographics and lesion details.

	Gender	Age (years)	MMSE	FMA score	MIQ-RS	Time of onset (days)	Type of stroke	Lesion volume (mm^3^)	Lesion location
1	Female	48	24	46	38	47	Hemorrhagic	22650	Basal ganglia
2	Man	27	25	27	46	70	Ischemic	11290	Basal ganglia
3	Man	65	28	45	42	70	Ischemic	2134	Striatum
4	Man	50	26	30	36	65	Hemorrhagic	814	Basal ganglia
5	Man	60	27	45	37	60	Hemorrhagic	3478	External capsule, striatum
6	Man	46	27	40	35	60	Hemorrhagic	6719	Basal ganglia
7	Man	59	29	42	30	60	Hemorrhagic	6352	Thalamus
8	Man	43	28	37	45	56	Hemorrhagic	8150	Thalamus
9	Female	50	26	30	39	40	Ischemic	3450	Basal ganglia
10	Man	58	30	28	30	74	Ischemic	12670	Thalamus

**Table 2:** The activated brain areas during motor execution and motor imagery in the two groups.

	Side	ME			*t* value	Voxels	MI			*t* value	Voxels
		*x*	*y*	*z*			*x*	*y*	*z*		
Control anatomic site											
M1	L	−36	−26	64	15.18	223	−50	−9	43	7.96	134
M1	R	58	−15	28	9.24	333	51	−9	50	6.01	114
PMC	L	−36	−21	60	13.48	891	−32	−5	58	6.63	672
PMC	R	40	−5	45	7.42	760	46	−3	51	5.31	670
SMA		−6	−3	58	11.70	648	−8	−0	57	7.90	686
Patients											
M1	L	−54	−24	28	6.15	75	−51	−11	20	6.52	110
M1	R	52	−24	44	4.59	110	54	−20	30	3.86	82
PMC	L	−24	−24	61	7.79	143	−37	1	54	5.07	134
PMC	R	38	−24	60	3.49	239	42	−5	46	3.17	125
SMA		−4	4	50	5.18	525	−2	19	62	5.54	479
